# Delivery of antisense oligonucleotide using polyethylenimine-based lipid nanoparticle modified with cell penetrating peptide

**DOI:** 10.1080/10717544.2019.1667453

**Published:** 2019-09-23

**Authors:** Shuang Yang, Dandan Wang, Yaojun Sun, Bin Zheng

**Affiliations:** a School of Basic Medical Sciences, Shanxi Medical University, Taiyuan, China;; b Affiliated Hospital, Changchun University of Chinese Medicine, Changchun, China;; c School of Pharmacy, Shanxi Medical University, Taiyuan, China

**Keywords:** Polyethylenimine, cell penetrating peptide, lipid nanoparticles, antisense oligonucleotide, cancer

## Abstract

Efficient and stable delivery system of antisense oligonucleotide (ASO) is important and urgently needed. Here, an ASO delivery system, Lp-PPRP, which contains a cationic polymer based on PEI (branched, 25 kDa), named PEI-PC and a palmitic acid modified R8 (R8-PA) was prepared to deliver a kind of ASO, LOR-2501. The characteristics of the nanoparticles and the cellular uptake of LOR-2501 in HeLa cells and A549 cells were studied. Lp-PPRP showed suitable particle size and zeta potential to combine with LOR-2501; the particle size and zeta potential of Lp-PPRP/LOR were 276.87 ± 5.63 nm and 18.03 ± 0.25 mV. *In vitro* experiments suggested that Lp-PPRP had lower cytotoxic and higher transfection efficiency for delivering LOR-2501 compared with PEI. The addition of PEI-PC and R8-PA contributed to enhance the transfection efficiency of the nanoparticles. In HeLa cells and A549 cells, Lp-PPRP could transport LOR-2501 and down-regulate the level of R1 protein efficiently, and the R1 down regulations were 64.56% and 66.34%, respectively. Results suggested potential utility of Lp-PPRP in the development of ASO in tumor therapy.

## 1. Introduction

The technology of antisense oligonucleotide (ASO) has been widely used in the field of tumor therapy because it can inhibit the processes of transcription and translation specifically (Wang et al., 2017; Golshirazi et al., 2018; Luna Velez et al., 2019; Raven et al., 2019). LOR-2501 is an ASO (20-mer phosphorothioate) targeting the mRNA of R1, which is a component of ribonucleotide reductase (RNR). Hence, R1 is an important target for the development of anti-tumor drugs (Yang et al., 2015b; Wu et al., 2018b). However, in the application of ASO, some challenges, such as poor stability, weak affinity with cell membrane and poor targeting still existed. Therefore, efficient and stable delivery system is an effective solution (Yang et al., 2014; Cheng et al., 2017; Mori et al., 2018).

Cationic polymer is a kind of promising carrier, such as polyethyleneimine (PEI), which contains large amounts of amino and has a high buffer capacity, thus PEI has strong agglomeration effect on ASO (Zhu et al., 2018; Mendes et al., 2019). PEI has a unique characteristic which is called as ‘proton sponge’ effect. It is conducive to the escape of the nanoparticles in the endosome and the drug release. However, high toxicity and low transfection efficiency limit the application of PEI (Zheng et al., 2019). The modification of PEI and the construction of its transmission system are important. Previous researches have indicated that hydrophobic modification was an effective means to improve the transfection efficiency and reduce the toxicity of PEI (Xie et al., 2013; Zheng et al., 2016; Dube et al., 2017; Feng et al., 2017).

Cell penetrating peptides (CPPs) have been shown to be effective in promoting siRNA, ASO, or nanoparticles to cross over cell membranes (Majumder et al., 2018; Singh et al., 2018; Srimanee et al., 2018). Octa-arginine (R8) is a classical cationic oligomeric CPP with high membrane penetrating efficiency. R8 has been successfully used in the delivery of siRNA, imaging agents or proteins (Zhang et al., 2006; Nakamura et al., 2007; Song et al., 2018; Wu et al., 2018a). It has been reported that increase the lipophilicity of CPPs could help to enhance the cell penetration efficiency (Huang et al., 2016; Lehto et al., 2017). Therefore, hydrophobic-modified PEI should provide excellent characteristics for the nanoparticles, such as stronger binding degree of ASO, lower toxicity, and the promotion of the endosomal escape and drug release into cytoplasm. While the lipophilicity of CPPs would be beneficial to improve the affinity between nanoparticles and the cell membrane of cancer cells, the modification could help to reduce the toxicity of CPPs. The co-existence of hydrophobic modified PEI and CPPs in the nanoparticles could bring out the synergistic effect; thus the obtained vector could have excellent characteristics of both PEI and CPPs, and it might have better protection and combination of ASO, safety and low toxicity, better stability, and better transfection effect.

**Figure 1.**
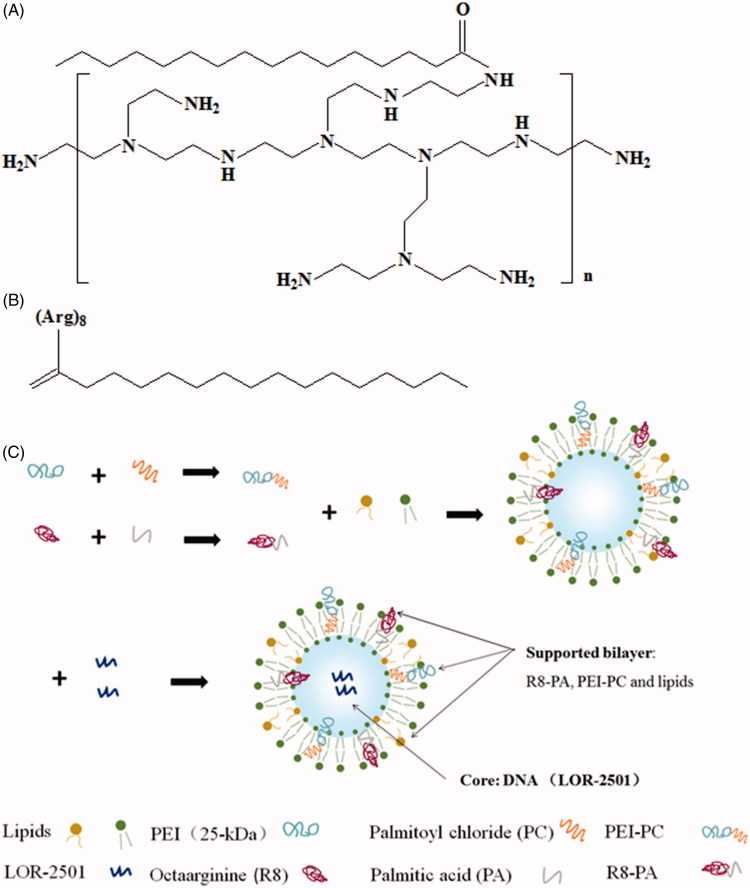
The structural formulae of PEI-PC, R8-PA, and schematic diagram of the preparation process of Lp-PPRP/LOR. (A) The structural formulae of PEI-PC. (B) The structural formulae of R8-PA. (C) The schematic diagram of the preparation process of Lp-PPRP/LOR.

In this report, a cationic polymer based on PEI (branched, 25 kDa), named PEI-PC and a palmitic acid modified R8 (R8-PA) were synthesized. PEI-PC and R8-PA were both used to prepare lipid nanoparticles, which were used to deliver a kind of ASO, LOR-2501 (Figure 1). LOR-2501 was wrapped in the core, PEI-PC, R8-PA, and lipids were incorporated into the supported bilayer of the nanoparticles. The characteristics of the nanoparticles and the cellular uptake of LOR-2501 complex in HeLa cells and A549 cells were studied.

## 2. Materials and methods

### 2.1. Materials

PEI (branched, 25 kDa), palmitic acid (PA) (≥99%), and palmitoyl chloride (98%) were purchased from Sigma-Aldrich (St. Louis, MO). Fmoc-l-arginine and 2-chlorotrityl chloride resins were purchased from JiEr Biochemistry Company (Shanghai, China). O-benzotriazol-1-yl-tetramethyluronium hexafluorophosphate (HBTU), 1-hydroxybenzotriazole (Hobt), and N,N-diisopropyl ethylamine (DIEA) were purchased from Xiya Chemical Technology Co., Ltd. (Chengdu, China). Egg phosphatidylcholine (ePC) and cholesterol were purchased from Avanti Polar Lipids, Inc. (Shanghai, China). LOR-2501 (5′-CTC TAG CGT CTT AAA GCC GA-3′) and 5′-Cy3-labled LOR-2501 were synthesized by Biomics Biotechnologies (Jiangsu, China). 4′,6-Diamidino-2-phenylindole (DAPI) was purchased from Thermo Fisher Scientific (Rockford, IL). Roswell Park Memorial Institute 1640 (RPMI 1640), Dulbecco’s modified Eagle’s medium (DMEM) and fetal bovine serum (FBS) were purchased from HyClone (Logan, UT). GAPDH, anti-R1 antibody and horseradish peroxidase (HRP)-conjugated goat anti-rabbit IgG were obtained from Abcam Inc. (Cambridge, MA). HeLa cells and A549 cells were purchased from American Type Culture Collection (ATCC). All other analytical reagents were commercially obtained in reagent grade.

### 2.2. Synthesis of PEI-PC and R8-PA

PEI-PC was synthesized by referring to the previous reports (Yang et al., 2015a, 2015c). First, 2 μmol PEI (branched, 25 kDa) was dissolved in 2 ml dichloromethane under N_2_ at room temperature (25 °C) and 2 μl triethylamine was added to the PEI solution. Then, the palmitoyl chloride was dissolved in 2 ml dichloromethane and gradually added to the obtained PEI solution at a palmitoyl chloride:ethylenimine molar ratio of 1:1.5. The reacted solution was stirred for 12 h under N_2_. The precipitate was washed by excess of ethyl ether for three times. Finally, the product was dried under vacuum overnight at room temperature (25 °C) and the power was stored in an airtight environment at 4 °C.

R8-PA was synthesized by using standard Fmoc solid phase methodologies as reported previously (Li et al., 2015, 2016). Briefly, Fmoc-l-arginine was assembled on a 2-chlorotrityl chloride resin to synthesize octa-arginine (R8). Then, PA was coupled to R8 at an R8:CA:HOBT:HBTU:DIEA molar ratio of 1:3:3:3:6. The product was purified by reverse-phase high-performance liquid chromatography (RP-HPLC) with a C18 column and using a linear gradient of ultrapure water and acetonitrile containing 0.1% trifluoroacetic acid. The product was dried under vacuum until into white or light yellow powder, sealed and stored in –20 °C. The structural formulae of PEI-PC and R8-PA are shown in Figure 1(A,B).

### 2.3. Preparation and characterization of the nanoparticles

The liposomes (Lp) or the liposomes containing LOR-2501 (Lp/LOR) were prepared by using an ethanol dilution method. For the Lp containing PEI-PC (Lp-PP), PEI-PC, ePC, and cholesterol were dissolved in ethanol at the molar ratio of 45:20:35 with homogeneous mixing. The obtained lipid solution was injected into the PBS buffer (pH 6.8) under rapid stirring at the volume ratio of 1:9 to obtain Lp-PP. For the Lp-PP containing LOR-2501 (Lp-PP/LOR), LOR-2501 was separately dissolved in DEPC water and added into the Lp-PP followed by vortexing for 30 s at various N/P ratios, which were from 2:1 to 12:1. Here, ‘N’ represents the molar weight of nitrogen in PEI-PC and ‘P’ represents the molar weight of phosphorus in LOR-2501. For the Lp containing PEI-PC and R8-PA (Lp-PPRP), PEI-PC, R8-PA, ePC, and cholesterol were dissolved in ethanol at R8-PA/total lipid ratios from 5% to 30%, respectively. LOR-2501 was dissolved in DEPC water and added into the Lp-PPRP followed by vortexing for 30 s at an optimal N/P ratio, the obtained Lp was named Lp-PPRP/LOR. Finally, all the particles were concentrated by ultrafiltration with a tubular polysulfone ultrafiltration membrane (MWCO 100 kDa). The schematic of the course preparation of the Lp is shown in Figure 1(C).

The particle sizes and zeta potentials of Lp-PP/LOR at various N/P ratios or Lp-PPRP/LOR at different R8-PA/total lipid ratios were determined on a Zetasizer Nano ZS 90 Instrument (Malvern Instruments, Ltd., Malvern, UK), respectively. Each formulation was calculated averaging three measurements. The Lp-PPRP/LOR was observed by field emission scanning electron microscope (FE-SEM) (JSM-6700F, JEOL, Tokyo, Japan) at 3.0 kV accelerating voltage.

### 2.4. Cell culture

HeLa cells were grown and propagated in DMEM; A549 cells were grown and propagated in RPM1640 supplemented with 10% FBS and 1% antibiotics (100 units/ml penicillin and 100 μg/ml streptomycin) at 37 °C in a humidified atmosphere containing 5% CO_2_.

### 2.5. Determination of the nanoparticles combined with LOR-2501 by gel retardation assay

Lp-PEI/LOR, Lp-PP/LOR at various N/P ratios, and Lp-PPRP/LOR were prepared. The complexes were confirmed by a gel retardation assay to measure the ability of LOR-2501 to form complexes with the liposomes. Ten microliter of the samples were incubated with 6× sample buffer at room temperature for 30 min and then loaded onto a 3% agarose gel containing 0.2% mg/ml ethidium bromide. Electrophoresis was performed at 120 V for 15 min. Finally, the gels were photographed under UV-illumination to observe the combination of the liposomes with LOR-2501.

### 2.6. Cytotoxicity assay of the polymers and the liposomes

For evaluating the viability of HeLa cells and A549 cells after they were treated with PEI, PP, Lp-PP, and Lp-PPRP. HeLa cells and A549 cells were seeded at a density of 1 × 10^4^ cells/well in a 96-well plate for 24 h containing 10% FBS. Cells were washed for three times with PBS buffer and then 100 μl of PEI, PP, Lp-PP, and Lp-PPRP in DMEM or RPM1640 medium without FBS were added at varying concentrations from 1 μg/ml to 10 μg/ml. The medium was removed after incubation for 4 h and the cells were incubated in fresh DMEM or RPM1640 medium for 20 h. For testing the cell vitalities, each well was incubated with 20 μl MTT solutions (5 mg/ml in PBS buffer) for 4 h. The medium was removed and then 100 μl/well of DMSO was added after removing the medium to dissolve the formed formazan crystals. The optical density was obtained by measuring at OD 570 nm in a plate reader. The results were summarized into % viability as mean ± SD of six replicates for each sample.

### 2.7. *In vitro* treatment and cellular uptake of the nanoparticles

The cellular uptakes of 5′-Cy3-labeled LOR-2501 liposomes were determined in HeLa and A549 cells by an EPICS XL flow cytometer (Beckman Coulter Corp., Tokyo, Japan). First, HeLa and A549 cells were plated on 24 well plates (1 × 10^5^ cells/well) and cultured overnight in 1 ml of DMEM or RPM1640 medium with 10% FBS. Then, PBS was added to wash the cells for three times and fresh serum-free medium was replaced. LOR-2501 labeled with 5′-Cy3 was formulated in Lp-PEI/LOR, Lp-PP/LOR, and Lp-PPRP/LOR. HeLa and A549 cells were treated with the above nanoparticles for 4 h at 37 °C. Then, the cells were washed with PBS for three times and fixed in 4% paraformaldehyde solution for 24 h at 4 °C. The cells were collected and analyzed by flow cytometry. Untreated cells were collected as a negative control. The mean fluorescence intensities of the cells were tested by three serial measurements at minimum.

### 2.8. Cellular internalization analysis of the nanoparticles by confocal microscopy

HeLa cells were cultured in a glass bottom cell culture dish at a density of 1 × 10^5^ cells per well for 24 h. LOR-2501 labeled with 5′-Cy3 was formulated in Lp-PEI/LOR, Lp-PP/LOR, and Lp-PPRP/LOR. HeLa cells were treated with naked LOR-2501, Lp-PEI/LOR, Lp-PP/LOR, and Lp-PPRP/LOR for 4 h at 37 °C. HeLa cells were washed for three times with PBS and fixed in 4% paraformaldehyde for 10 min. For observing the cellular internalization of the nanoparticles, cellular nuclei were stained with DAPI (2.5 μg/ml) for 3 min. The cells were washed for three times with PBS before the observation. The internalizations were observed by Zeiss 710 LSMNLO Confocal Microscope (Carl Zeiss, Jena, Germany).

### 2.9. Determination of R1 protein expression after treating with the nanoparticles

HeLa and A549 cells were plated on six well plates (1 × 10^5^ cells/well) for 24 h. Then, the medium was removed and cells were treated with Lp-PEI/LOR, Lp-PP/LOR, and Lp-PPRP/LOR for 4 h. The medium containing the nanoparticles was removed and the cells were cultured for another 44 h. RIPA buffer (Sigma, St. Louis, MO) supplemented with 2% PMSF and 1% protease inhibitor cocktail (Sigma, St. Louis, MO) was added to each well to lyse the cells for 10 min in an ice-cold bath. The protein solutions were collected and centrifuged at 10,000 rpm for 5 min. A portion of the supernatant was used for protein concentration determination using the Bradford protein assay. The other protein samples were mixed with the loading buffer and heated for 6 min at 95 °C. Then, samples (30 μg total proteins) were separated by 10% SDS-PAGE gel and transferred onto polyvinylidene fluoride (PVDF) membrane. The PVDF membrane was blocked with 5% bovine serum albumin for 3 h and immunoblotted against GAPDH or anti-R1 antibody at 4 °C overnight. Next, the membrane was incubated with HRP-conjugated goat anti-rabbit IgG for 4 h at 4 °C. ECL detection kits (GE Healthcare Life science, Waukesha, WI) were used for detecting the chemiluminescence.

### 2.10. Statistical analysis

All the data were expressed as mean ± standard deviation (SD). The identification of significant differences between groups was carried out with *t*-test. *p* < .05 represents significant difference, and *p* < .01 indicates a highly significant difference.

## 3. Results

### 3.1. Formulation optimization of Lp-PP/LOR and Lp-PPRP/LOR

**Figure 2.**
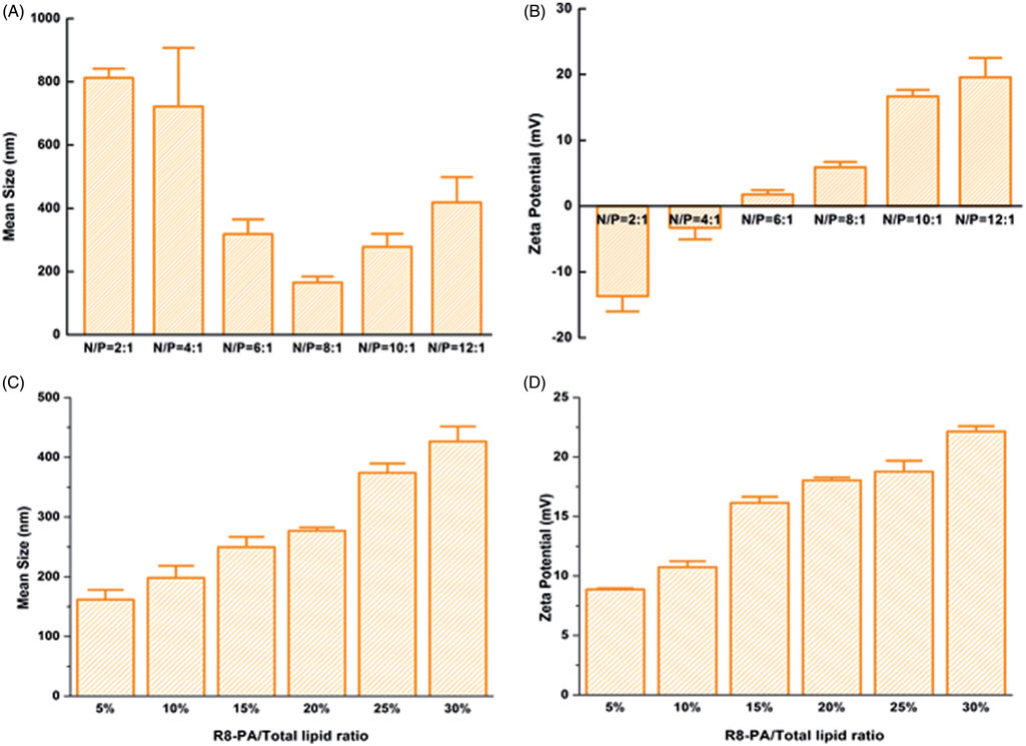
Mean sizes and zeta potentials of Lp-PP/LOR and Lp-PPRP/LOR. (A) Mean sizes of Lp-PP/LOR at varying N/P ratios from 2:1 to 12:1. (B) Zeta potentials of Lp-PP/LOR at varying N/P ratios from 2:1 to 12:1. (C) Mean sizes of Lp-PPRP/LOR at varying R8-PA/total lipid ratios from 5% to 30%. (D) Zeta potentials of Lp-PPRP/LOR at varying R8-PA/total lipid ratios from 5% to 30%. ‘N’ represented the molar weight of nitrogen in PP and ‘P’ represented the molar weight of phosphorus in LOR.

The particle sizes and zeta potentials of Lp-PP/LOR at various N/P ratios and Lp-PPRP/LOR at R8-PA/total lipid ratios were measured (Figure 2). The influence of different N/P ratios on the particle sizes of Lp-PP/LOR is shown in Figure 2(A). When N/P ratios changed from 2:1 to 12:1; particle sizes were stable in the range of 6:1 to 10:1. When the ratios were less than 6:1, the particle sizes were large and unstable. When the N/P ratio was larger than 10:1 (12:1), the particle size tended to increase. For the zeta potentials of Lp-PP/LOR at various N/P ratios, the data are shown in Figure 2(B). When the N/P ratios were greater than 6:1, the zeta potentials changed from negative to positive. The increase of the zeta potentials was not obvious when the ratios were greater than 8:1. According to the optimization results of the particle sizes and zeta potentials of Lp-PP/LOR, N/P = 8:1 was the optimal proportion, and the particle size and zeta potential under this condition were 165.23 ± 19.05 nm and 5.89 ± 0.80 mV, respectively.

The influence of R8-PA/total lipid ratios on the particle sizes and zeta potentials of Lp-PPRP/LOR is shown in Figure 2(C,D). The increase in R8-PA increased the particle sizes and zeta potentials gradually. When R8-PA/total lipid ratio was 20%, the particle size and zeta potential were relatively stable and appropriate, and the values were 276.87 ± 5.63 nm and 18.03 ± 0.25 mV, respectively. So, the optimal proportion was R8-PA/total lipid ratio = 20% from the optimization of particle size and zeta potential.

### 3.2. Agarose gel electrophoresis retardation assay of Lp-PP/LOR and Lp-PPRP/LOR

**Figure 3.**
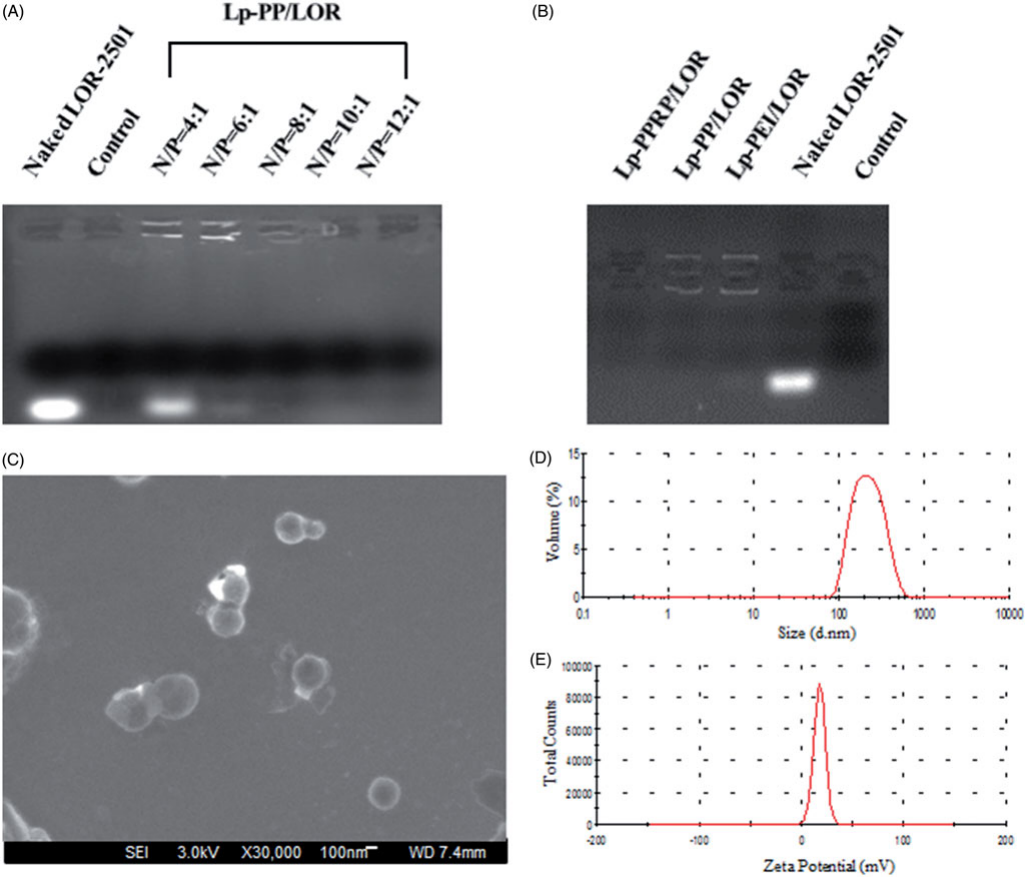
Agarose gel electrophoresis retardation assay of Lp-PP/LOR, Lp-PPRP/LOR and the characterization of Lp-PPRP/LOR. (A) Agarose gel electrophoresis retardation assay of Lp-PP/LOR at varying N/P ratios. (B) Agarose gel electrophoresis retardation assay of Lp-PPRP/LOR. (C) FE-SEM image of Lp-PPRP/LOR at R8-PA/total lipid ratio = 20%. (D) Diameter distribution of Lp-PPRP/LOR at R8-PA/total lipid ratio = 20%. (E) Zeta potential distribution of Lp-PPRP/LOR at R8-PA/total lipid ratio = 20%.

Agarose gel electrophoresis retardation assay was investigated to observe the combination of the vectors and LOR-2501. As shown in Figure 3(A,B), for Lp-PP/LOR at various N/P ratios, Lp-PP combined with a part of LOR-2501 when the N/P ratio was 4:1; the bright banding of LOR-2501 decreased. As the N/P ratio increased, the bright banding disappeared, which showed that Lp-PP could completely combine with LOR-2501 when the N/P ratio was greater than 6:1. Figure 3(B) shows that Lp-PEI and Lp-PP could completely combine with LOR-2501 at the N/P ratio = 8:1, and Lp-PPRP could also completely retard to LOR-2501 to form particle at R8-PA/total lipid ratio = 20%. Hence for the result of the agarose gel electrophoresis retardation assay, N/P ratio = 8:1 and R8-PA/total lipid ratio = 20% were the appropriate ratios for Lp-PP/LOR and Lp-PPRP/LOR, respectively. Lp-PP and Lp-PPRP could effectively load LOR-2501 and the obtained nanoparticles could be used for subsequent evaluation experiments.

### 3.3. Characterization of Lp-PPRP/LOR

In order to observe the morphology and size of the formed Lp-PPRP/LOR visually, Lp-PPRP/LOR at R8-PA/total lipid ratio = 20% was observed under FE-SEM; the result is shown in Figure 3(C). The FE-SEM image showed that the morphology of Lp-PPRP/LOR was intact; the surface was smooth and spherical; the particle size of Lp-PPRP/LOR was uniform (200–300 nm) within the field of view, which was consistent with the values of particle size experiment. Figure 3(D,E) shows the diameter distribution and the zeta potential distribution of Lp-PPRP/LOR at the R8-PA/total lipid ratio of 20% were 276.87 ± 5.63 nm and 18.03 ± 0.25 mV, respectively.

### 3.4. Cytotoxicity tests

**Figure 4.**
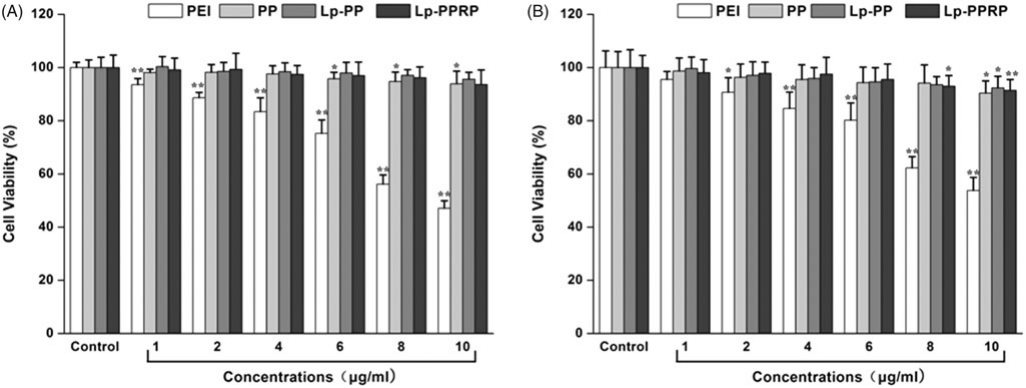
Cytotoxicity tests of PEI, PP, LP-PP and LP-PPRP. (A) Cytotoxicity tests of PEI, PP, LP-PP, and LP-PPRP on HeLa cells. (B) Cytotoxicity tests of PEI, PP, LP-PP, and LP-PPRP on A549 cells. Each bar is the mean of six experiments normalized to mean ± SD. *p < .05 vs. control and **p < .01 vs. control.

MTT assay was used to test the viability of HeLa cells and A549 cells after they were treated with different concentrations (in terms of the concentration of the polymers) of PEI, PP, Lp-PP, and Lp-PPRP; the results are shown in Figure 4(A,B). In HeLa cells, for the group of PEI, the cell viabilities decreased gradually with the increase of the concentration of PEI. When the concentration achieved 10 μg/ml, the cell viability of HeLa cells was only 47.07%. Compared with PEI, PP showed lower toxicity, the cell viabilities remained above 90% at the tested concentrations. Lp-PP and Lp-PPRP also showed no obviously toxicity in HeLa cells. In A549 cells, for the groups of PP, Lp-PP, and Lp-PPRP, the cell viabilities were not significantly affected compared with PEI, which showed PP, Lp-PP, and Lp-PPRP were safe as the vehicles for LOR-2501.

### 3.5. Cellular uptakes of the nanoparticles determined by flow cytometry

**Figure 5.**
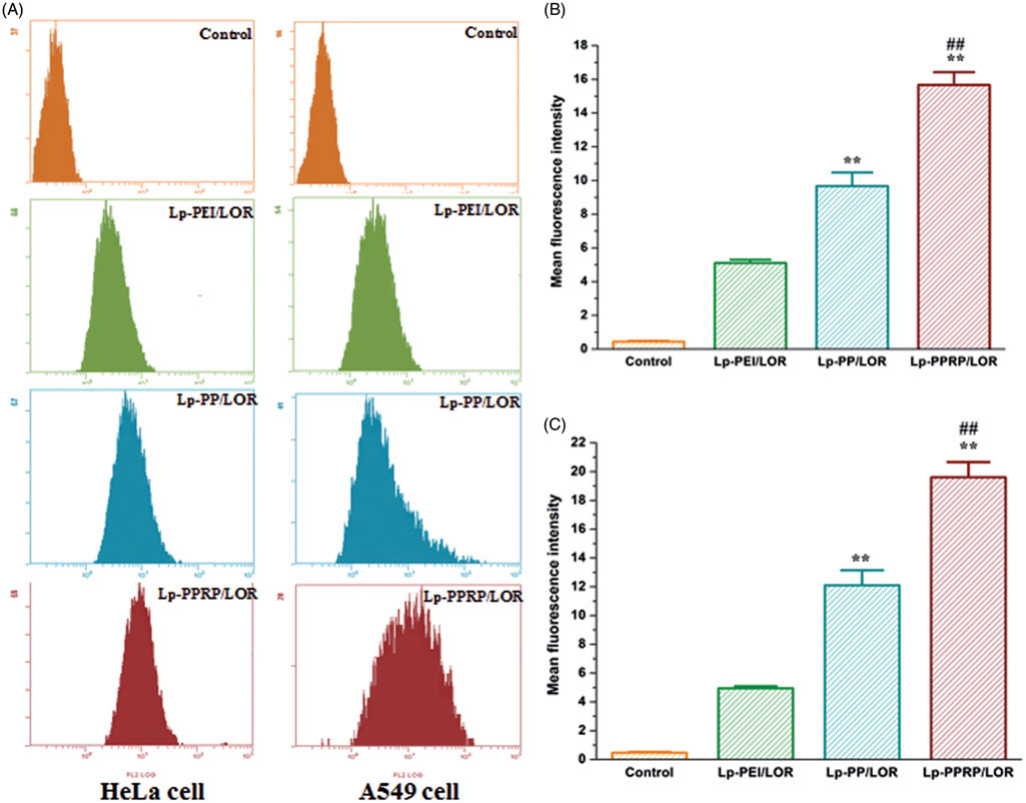
Cellular uptake of 5′-Cy3-labled LOR-2501 delivered by Lp-PEI, Lp-PP, and Lp-PPRP. (A) Cellular fluorescence uptake of Lp-PEI/LOR, Lp-PP/LOR, and Lp-PPRP/LOR by flow cytometry in HeLa cells and A549 cells. (B) The mean fluorescence values of HeLa cells treated with Lp-PEI/LOR, Lp-PP/LOR, and Lp-PPRP/LOR. (C) The mean fluorescence values of A549 cells treated with Lp-PEI/LOR, Lp-PP/LOR, and Lp-PPRP/LOR. LOR-2501 was labeled with 5′-Cy3. **p < .01 vs. Lp-PEI/LOR and ^##^*p* < .01 vs. Lp-PP/LOR.

Cellular uptakes of 5′-Cy3-labled LOR-2501 delivered by Lp-PEI, Lp-PP, and Lp-PPRP in HeLa cells and A549 cells were determined by flow cytometry; the results are shown in Figure 5. The fluorescence transfer curves in HeLa cells and A549 cells (Figure 5(A)) showed that the curves of Lp-PEI, Lp-PP, and Lp-PPRP significantly migrated compared with the group of control, and the migration of Lp-PPRP was more obvious than Lp-PEI, Lp-PP both in HeLa cells and A549 cells. The mean fluorescence intensities of HeLa cells and A549 cells treated with Lp-PEI, Lp-PP, and Lp-PPRP are shown in Figure 5(B,C), respectively. The mean fluorescence intensity values of the group of Lp-PPRP/LOR were 15.67 ± 0.76 and 19.60 ± 1.06 in HeLa cells and A549 cells, which were more than three times that of Lp-PEI/LOR and 1.5 times that of Lp-PP/LOR. For the cells treated with Lp-PPRP/LOR, the mean fluorescence intensity was significantly increased compared with Lp-PEI/LOR and Lp-PP/LOR (*p* < .01), which showed Lp-PPRP was effective in improving the transfection efficiency of LOR-2501.

### 3.6. Determination of R1 protein expression

**Figure 6.**
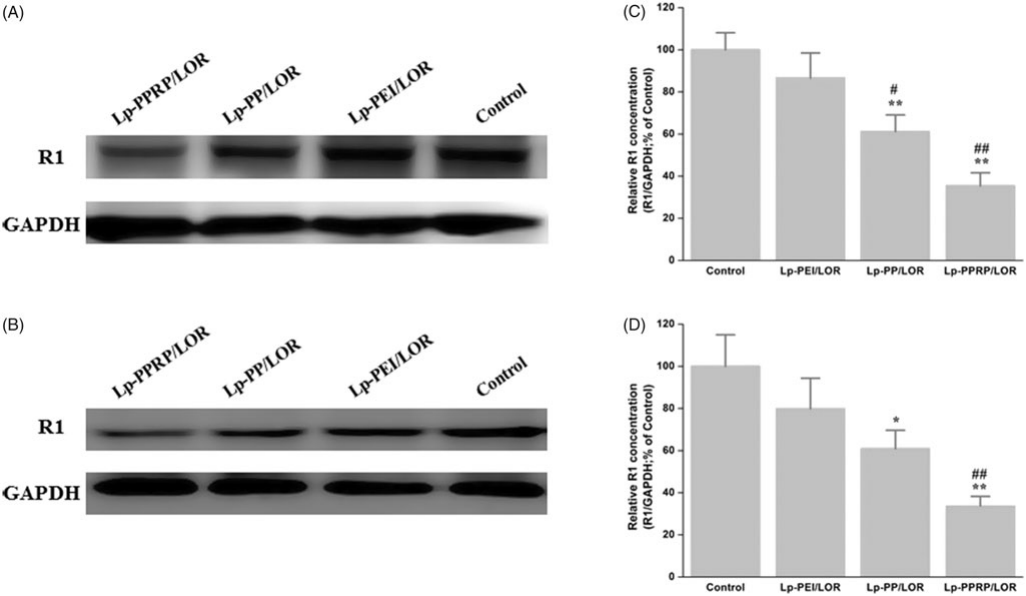
Down regulation of R1 protein expression in HeLa cells and A549 cells treated with Lp-PEI/LOR, Lp-PP/LOR, and Lp-PPRP/LOR. (A, C) HeLa cells. (B, D) A549 cells. *p < .05 vs. Lp-PEI/LOR, **p < .01 vs. Lp-PEI/LOR, ^#^*p* < .05 vs. Lp-PP/LOR, and ^##^*p* < .01 vs. Lp-PP/LOR.

The R1 protein levels in HeLa cells and A549 cells treated with Lp-PEI/LOR, Lp-PP/LOR and Lp-PPRP/LOR were determined by western blot analysis; the results are shown in Figure 6. In HeLa cells, the R1 down regulations of Lp-PEI/LOR, Lp-PP/LOR, and Lp-PPRP/LOR were 13.48%, 38.84%, and 64.56%, respectively (Figure 6(A,C)). In A549 cells, the R1 down regulations of Lp-PEI/LOR, Lp-PP/LOR, and Lp-PPRP/LOR were 20.17%, 39.07%, and 66.34%, respectively (Figure 6(B,D)). Compared with Lp-PEI/LOR and Lp-PP/LOR, Lp-PPRP/LOR showed significant activity of R1 down regulation, which showed that the addition of R8-PA was effective for increasing the transfection efficiency of LOR-2501 in HeLa cells and A549 cells. The transmembrane effect of R8-PA facilitated the entry of PEI-based lipid nanoparticles into cancer cells.

### 3.7. Intracellular localization of 5′-Cy3-labled LOR-2501

In order to observe the transfection effect more directly, the transfections of naked LOR-2501, Lp-PEI/LOR, Lp-PP/LOR, and Lp-PPRP/LOR were observed by confocal laser microscopy. In the absence of the carriers, naked LOR-2501 showed weaker red fluorescence in the field of vision. It was mainly due to the strong negative charge of LOR-2501 and the degradation by nuclease. Naked LOR-2501 failed to concentrate on the surface of HeLa cells or cross the membrane into the cells in large quantities. Compared with the naked LOR-2501, the red fluorescence signals of the groups of Lp-PEI/LOR, Lp-PP/LOR, and Lp-PPRP/LOR were enhanced in the field of vision, especially the group of Lp-PPRP/LOR. 5′-Cy3-labled LOR-2501 delivered by Lp-PPRP showed strong fluorescence signal appeared around or inside HeLa cells. 5′-Cy3-labled LOR-2501 was mainly distributed in the cytoplasm, indicating that the prepared Lp-PPRP could transfect LOR-2501 into tumor cells and release LOR-2501 into the cytoplasm.

## 4. Discussion

Efficient ASO delivery system needs further optimization and improvement (Farooqi et al., 2014; Moss et al., 2019). In this study, PEI-based lipid nanoparticle modified with CPP was prepared. PEI-PC, a cationic polymer based on PEI and an R8-PA were used to prepare the lipid nanoparticles to deliver LOR-2501. The formulation optimization and characteristics of the nanoparticles containing LOR-2501 were investigated. A series of *in vitro* evaluation experiments were used to evaluate the transfection effect of the nanoparticles in HeLa cells and A549 cells.

The particle sizes and zeta potentials of Lp-PP/LOR and Lp-PPRP/LOR were measured to achieve appropriate N/P ratio and R8-PA/total lipid ratio. Results showed that N/P = 8:1 (Figure 2(A,B)) and R8-PA/total lipid ratio = 20% (Figure 2(C,D)) were the optimal proportion for Lp-PP/LOR and Lp-PPRP/LOR, respectively. The vectors with appropriate concentration had positive effect on the delivering of LOR-2501. Excessive concentration of the carrier can be detrimental to the formation of nanoparticles. Agarose gel electrophoresis retardation assay showed the total combination between Lp-PP and LOR-2501 at N/P ratio >6:1. Lp-PP and Lp-PPRP could effectively load LOR-2501 at appropriate ratios to form nanoparticles (Figure 3(A,B)). The particle size and zeta potential distribution of Lp-PPRP/LOR prepared by the optimized ratio were 276.87 ± 5.63 nm and 18.03 ± 0.25 mV, which were appropriate for ASO delivery. The suitable positive charge can effectively bind with the negative charged LOR-2501 (Figure 3(C,D)). Low toxicity is an important property of the vectors of ASO. Our results on the cytotoxicity of PEI, PP, Lp-PP, and Lp-PPRP showed that the cell viabilities of PP, Lp-PP, and Lp-PPRP were all above 90% at the tested concentrations in HeLa cells and A549 cells (Figure 4). All the above tests proved that Lp-PP and Lp-PPRP were the appropriate vectors for ASO.

**Figure 7.**
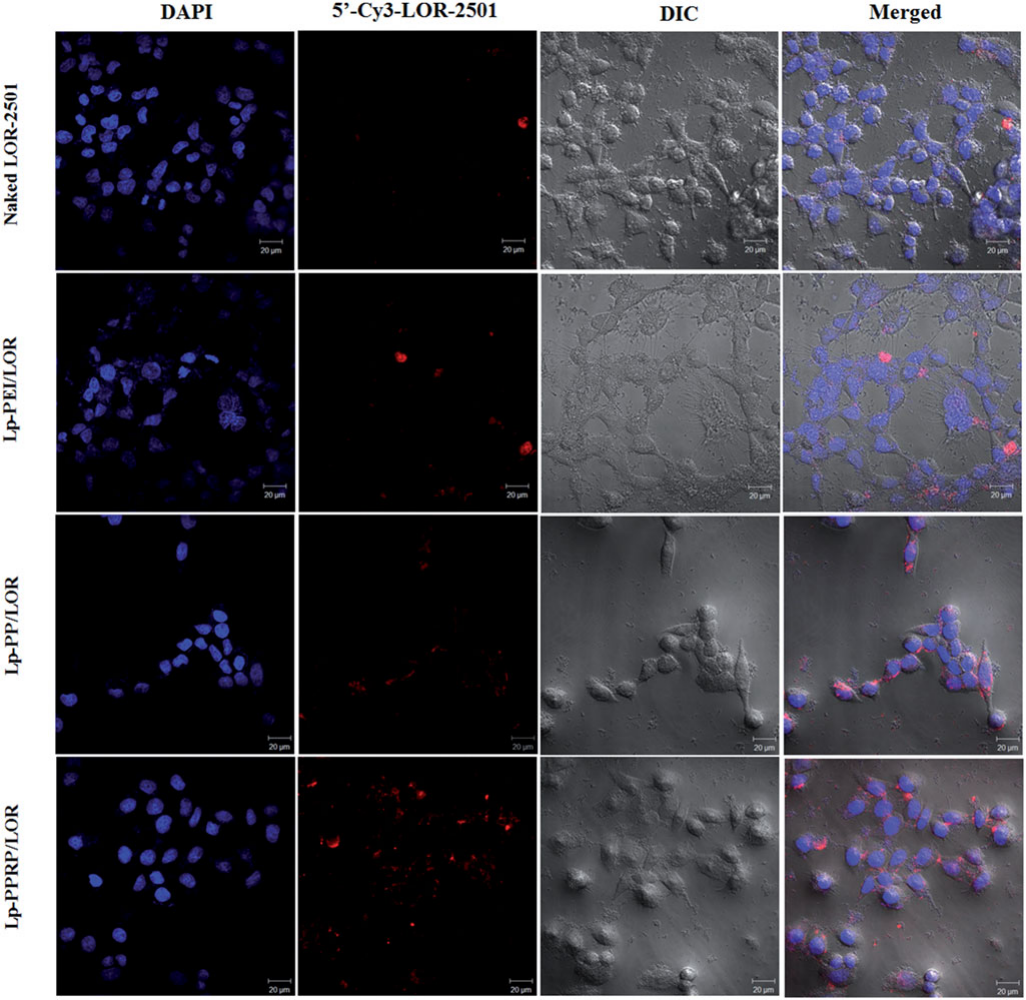
Intracellular localization of 5′-Cy3-labled LOR-2501 delivered by Lp-PEI, Lp-PP, and Lp-PPRP in HeLa cells shown by confocal microscopy. 5′-Cy3-labled LOR-2501 is shown in red, DAPI nuclear stain is shown in blue. Scale bar = 20 μm.

In order to evaluate the cellular uptakes of LOR-2501 delivered by Lp-PEI, Lp-PP, and Lp-PPRP in HeLa cells and A549 cells, flow cytometry was first used, results showed that the mean fluorescence intensity values of the group of Lp-PPRP/LOR were more than three times that of Lp-PEI/LOR and 1.5 times that of Lp-PP/LOR (Figure 5). Then, western blot analysis was used to determine the R1 protein levels in HeLa cells and A549 cells treated with Lp-PEI/LOR, Lp-PP/LOR, and Lp-PPRP/LOR, the R1 down regulations of Lp-PPRP/LOR were 64.56% and 66.34% in HeLa cells and A549 cells, respectively (Figure 6). The intracellular localization of 5′-Cy3-labled LOR-2501 delivered by Lp-PEI, Lp-PP, and Lp-PPRP was observed by confocal laser microscopy. The group of Lp-PPRP/LOR showed more extensive internalization than that of Lp-PEI/LOR and Lp-PP/LOR; the prepared Lp-PPRP could transfect 5′-Cy3-labled LOR-2501 into the cytoplasm of HeLa cells effectively (Figure 7). The above *in vitro* evaluation experiments showed that Lp-PPRP was a low toxicity and high efficiency vehicle of ASO. It might be due to the co-existence of PEI-PC and R8-PA in Lp-PPRP. The hydrophobic modification of PEI (PEI-PC) was beneficial to improve the affinity between nanoparticles and cell membrane and to promote the ASO release. The R8-PA reduced the toxicity and increased the transmembrane effect of the nanoparticles.

## 5. Conclusions

In conclusion, the lipid nanoparticles (Lp-PPRP) containing a cationic polymer based on PEI (PEI-PC) and an R8-PA were efficient to deliver a kind of ASO, LOR-2501. We showed that the Lp-PPRP was able to bind LOR-2501 efficiently. *In vitro* experiments demonstrated that the Lp-PPRP had much lower cytotoxicity and greater transfection activity compared to PEI. We suggested that Lp-PPRP might be suitable and effective for further *in vivo* study for the delivery of ASO.
